# The Use of Alloderm® Coverage to Reinforce Tissues in Two-Stage Tissue Expansion Placement in the Subcutaneous (Prepectoral) Plane: A Prospective Pilot Study

**DOI:** 10.7759/cureus.27680

**Published:** 2022-08-04

**Authors:** Rafael Felix P Tiongco, Joseph S Puthumana, Iman F Khan, Pathik Aravind, Michael A Cheah, Justin M Sacks, Michele Manahan, Carisa M Cooney, Gedge D Rosson

**Affiliations:** 1 Department of Plastic and Reconstructive Surgery, Johns Hopkins University School of Medicine, Baltimore, USA

**Keywords:** sf-36 questionnaire, breast-q questionnaire, pain on vas, prepectoral breast reconstruction, adm, tissue expander

## Abstract

Purpose: Two-stage tissue expander (TE) to implant breast reconstruction is commonly performed by plastic surgeons. Prepectoral implant placement with acellular dermal matrix (ADM, e.g., AlloDerm®) reinforcement is evidenced by minimal postoperative pain. However, the same is not known for TE-based reconstruction. We performed this study to explore the use of complete AlloDerm® reinforcement of breast pocket tissues in women undergoing unilateral or bilateral mastectomies followed by immediate, two-stage tissue expansion in the prepectoral plane.

Methods: Patients (n = 20) aged 18-75 years were followed prospectively from their preoperative consult to 60 days post-TE insertion. The pain visual analog scale (VAS), Patient Pain Assessment Questionnaire, Subjective Pain Survey, Brief Pain Inventory-Short Form (BPI-SF), postoperative nausea and vomiting (PONV) survey, BREAST-Q Reconstruction Module, and short-form 36 (SF-36) questionnaires were administered. Demographic, intraoperative, and 30- and 60-day complications data were abstracted from medical records. After TE-to-implant exchange, patients were followed until 60 days postoperatively to assess for complications.

Results: Pain VAS and BPI-SF pain interference scores returned to preoperative values by 30 days post-TE insertion. Static and moving pain scores from the Patient Pain Assessment Questionnaire returned to preoperative baseline values by day 60. The mean subjective pain score was 3.0 (0.5 standard deviation) with seven patients scoring outside the standard deviation; none of these seven patients had a history of anxiety or depression. Median PONV scores remained at 0 from postoperative day 0 to day 7. Patient-reported opioid use dropped from 89.5% to 10.5% by postoperative day 30. BREAST-Q: Sexual well-being scores significantly increased from preoperative baseline to day 60 post-TE insertion. Changes in SF-36 physical functioning, physician limitations, emotional well-being, social functioning, and pain scores were significantly different from preoperative baseline to day 60 post-TE insertion. Five participants had complications within 60 days post-TE insertion. One participant experienced a complication within 60 days after TE-to-implant exchange.

Conclusions: We describe pain scores, opioid usage, patient-reported outcomes data, and complication profiles of 20 consecutive patients undergoing mastectomy followed by immediate, two-stage tissue expansion in the prepectoral plane. We hope this study serves as a baseline for future research.

## Introduction

Breast reconstruction is typically performed using implants or autologous tissue transplantation. Though autologous transfer is considered the gold standard for restoration to match the original breast, particularly in irradiated patients, implant-based breast reconstruction remains the most widely used approach in the United States, and the case numbers continue to rise [1,2]. Implant-based reconstruction can be performed in either one or two stages. Single-stage reconstruction with immediate placement of a prosthetic implant enables patients to awaken from surgery with a reconstructed breast but largely relies on mastectomy flap quality. The more common two-stage approach involves the placement of a tissue expander (TE) to preserve the natural breast footprint followed by an exchange with a prosthetic implant once the skin envelope has been expanded to the desired volume [1,3].

Traditionally, two-stage breast reconstruction involved total submuscular coverage of the TE/implant beneath the pectoralis major and serratus anterior [4]. Though cosmetic results have been described to be excellent, total submuscular reconstruction is associated with significant postoperative pain, injury-induced muscular deficits, the potential for breast animation deformity (>70% of patients), lateral deviation of the breast mound with poor inframammary fold definition, and insufficient lower pole fullness [1,5-7]. In order to reduce the manipulation of the pectoralis, acellular dermal matrix (ADM) (e.g., AlloDerm®) emerged as an appropriate product to reinforce the poorly supported lower pole of the breast pocket. These dual plane reconstructions, also known as partial subpectoral or partial sub-ADM, avoid some of the complications described above by minimizing the elevation of the pectoralis [7]. Moreover, they offer comparable or better cosmetic outcomes, a similar safety profile, better early fill volumes, and less postoperative pain than submuscular reconstruction [4].

Prepectoral TE breast reconstruction with ADM presents an opportunity to improve upon the current reconstructive methods and minimize postoperative pain. However, little is known regarding the effectiveness of ADM in pain reduction using a two-stage reconstructive approach in the prepectoral plane [1,8]. We performed the current prospective study to explore the use of complete AlloDerm® reinforcement of breast pocket tissues in women undergoing mastectomies followed by two-stage reconstruction with tissue expansion and implant placement in the prepectoral plane.

## Materials and methods

Patient recruitment

This clinical trial (ClinicalTrials.gov<http://ClinicalTrials.gov> Identifier: NCT03195322) was approved by the Johns Hopkins Medicine Institutional Review Board (IRB00091477). Patients aged 18-75 years undergoing unilateral or bilateral mastectomies followed by immediate, TE placement in the prepectoral plane with complete AlloDerm® coverage were eligible for inclusion. Patients were excluded if they (1) underwent final reconstruction with autologous material, (2) were allergic to cefoxitin, lincomycin, vancomycin, or polymyxin, (3) had active connective tissue disease, or (4) were current smokers.

Surgical technique

**Figure 1:**
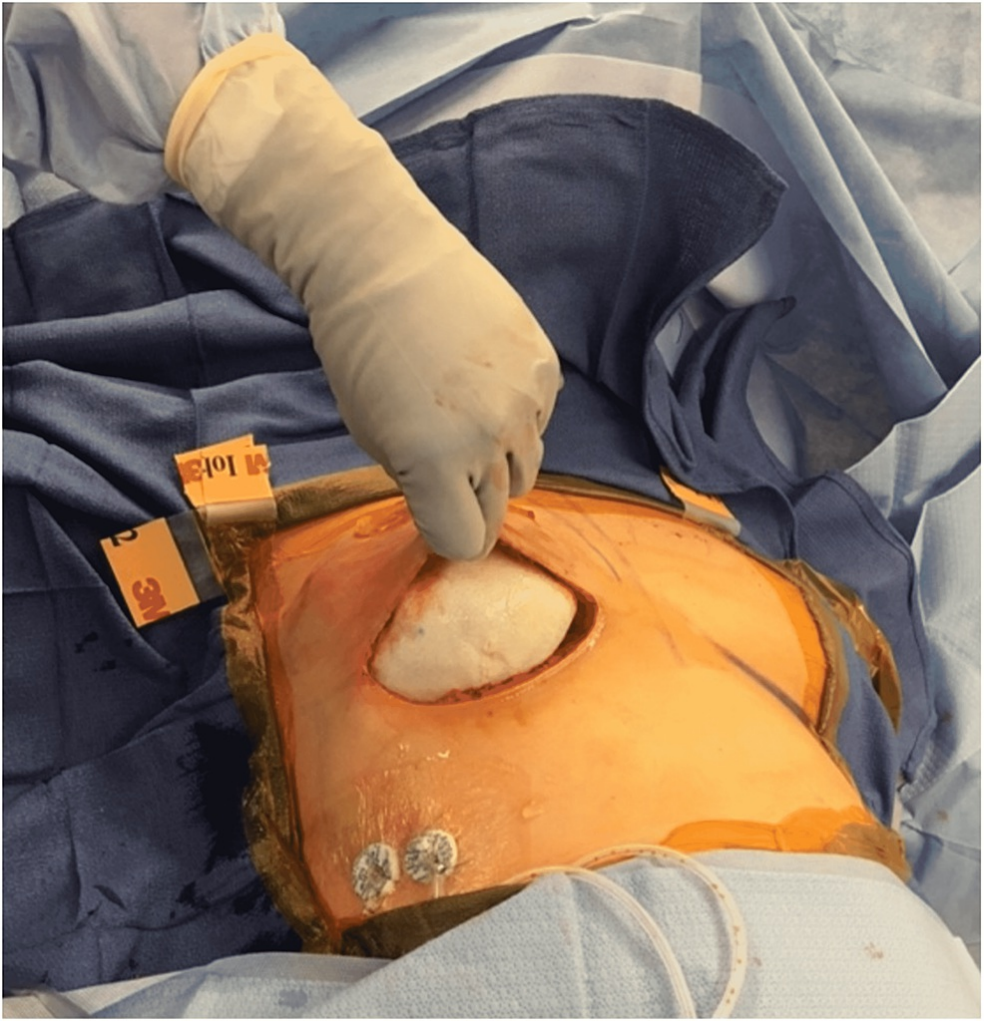
Intraoperative view of the tissue expander placed in a pocket

Surgical intervention for study participants followed the standard of care. After the surgical oncology team finished their portion of the procedure, the plastic surgeon evaluated the mastectomy skin flap to ensure the patient was a candidate for prepectoral placement of the TE. The TE was then soaked in an antibiotic solution and placed deep into the mastectomy skin flap using three suture tabs to secure them to the chest wall (Figure 1).

**Figure 2:**
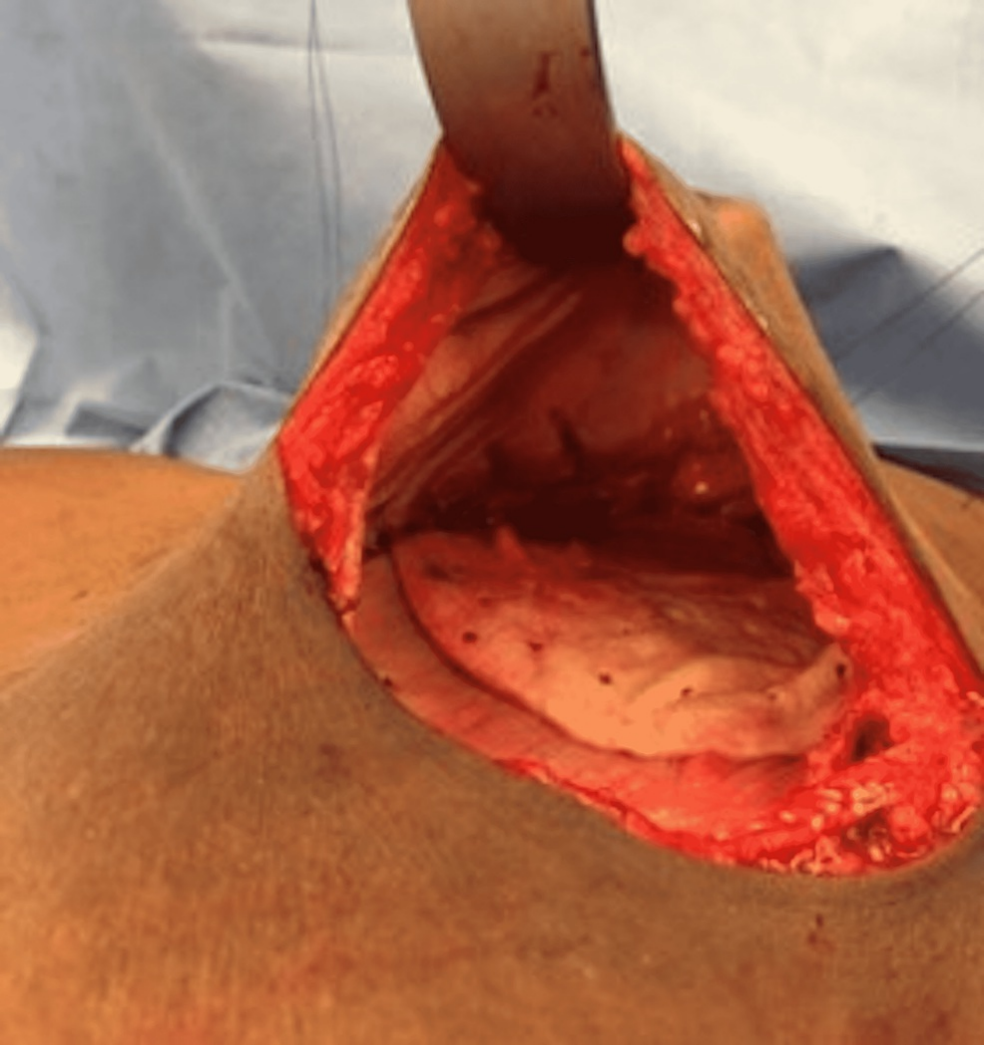
Intraoperative view of the breast pocket after removal of the tissue expander with Alloderm® seen on the surface of the pectoralis major

**Figure 3:**
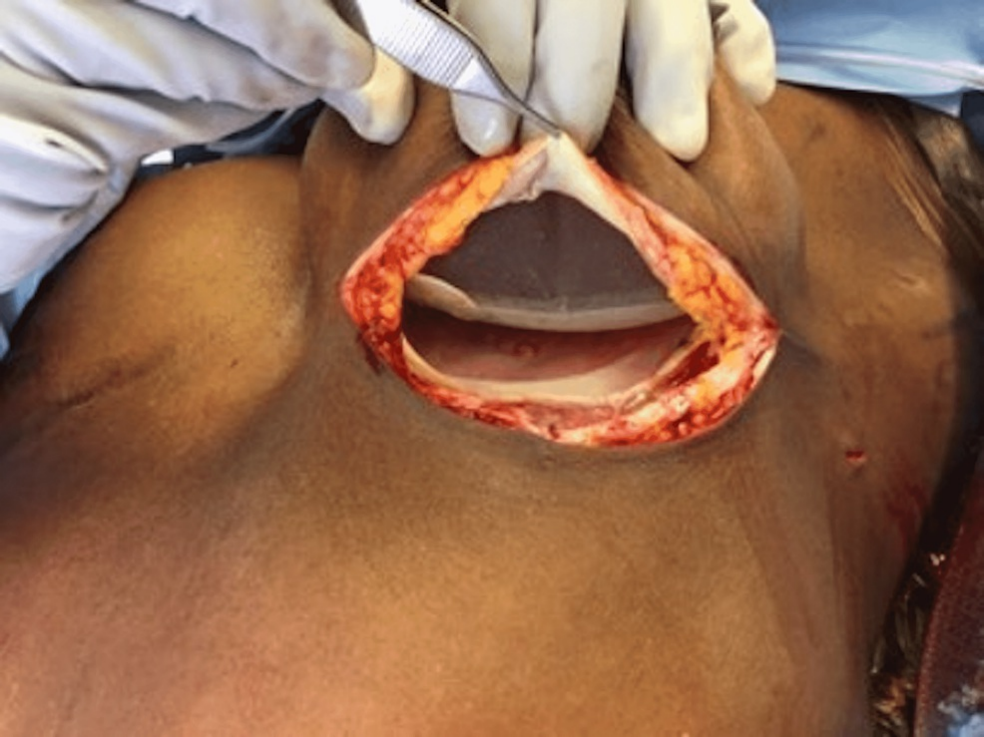
Intraoperative view of the permanent implant placed in a pocket

AlloDerm® was also soaked in an antibiotic solution and sewn into the skin flap over the TE to reinforce the pocket. The breast pocket was then irrigated with antibiotic solution, and the mastectomy skin defect was closed in layers. The procedure was completed for the contralateral breast if indicated. Following the full expansion of the TE, the second stage was performed. The TE was removed, and then the permanent implant was placed deep into the skin flap (Figures 2, 3).

Study assessments and data collection

Following informed consent, all participants were asked to complete patient-reported outcome questionnaires at their initial visit. These included the pain visual analog scale (VAS) [9], the Patient Pain Assessment Questionnaire, the Subjective Pain Survey, the preoperative BREAST-Q: Reconstruction Module [10], the short-form 36 (SF-36) [11], and the Brief Pain Inventory-Short Form (BPI-SF) [12] where patients can report pain medication usage. The Patient Pain Assessment Questionnaire and Subjective Pain Survey may be requested from authors as needed. Demographic, complications, and intraoperative data were abstracted from medical records.

On postoperative day 0, immediately following TE placement, the pain VAS, Patient Pain Assessment Questionnaire, BPI-SF, and postoperative nausea and vomiting (PONV) intensity scale were administered [13]. These surveys were readministered on postoperative days 1, 2, 3, and 7 ± 2. The SF-36 was administered on day 7 ± 2.

Long-term quality-of-life and health outcomes assessments were performed on postoperative days 30 ± 4 and 60 ± 7 post-TE placement. During these visits, pain scores (pain VAS, Patient Pain Assessment Questionnaire, and BPI-SF), postoperative BREAST-Q reconstructive module surveys [10], and SF-36 questionnaires were administered. Complications (seroma, hematoma, cellulitis, and infection requiring expander removal) that occurred within 60 days postoperatively were recorded. After TE-to-implant exchange, patients were followed until 60 days postoperatively to assess for complications.

Statistical analysis

Statistical analyses were performed using IBM Statistical Package for the Social Sciences (SPSS) software version 27 (IBM Corp., Armonk, NY). Data on pain VAS scores, static and moving pain scores from the Patient Pain Assessment Questionnaire, BPI-SF pain interference scores, opioid use, BREAST-Q scores, and SF-36 subscores were analyzed descriptively with median and interquartile range. Comparisons across timepoints for BREAST-Q and SF-36 scores were conducted by non-parametric Kruskal-Wallis test. Significance was assessed at a two-sided p-values < 0.05.

## Results

Patient demographic and intraoperative data

A total of 39 breasts in 20 consecutive female patients were reconstructed between October 2017 and December 2018. The mean age was 42 years (range: 29-56), and BMI was 25.1 (range: 12.8-35). The majority of patients were white (80%), married (75%), and did not have a history of smoking (85%). Anxiety (10%) and depression (15%) were reported in our patient cohort. Tumor data are contained in Table 1.

**Table 1:** Patient demographics IDC: Invasive ductal carcinoma; DCIS: Ductal carcinoma in situ; ILC: Invasive lobular carcinoma; IMC: Invasive mammary carcinoma; ALH: Atypical lobular hyperplasia; TNM: tumor, nodes, and metastases; BRCA: BReast CAncer gene.

Variable	Mean (SD)
Age	(41.7, 7.6)
BMI	(25.1, 4.7)
Race	Count
Asian	2
Black	1
Hispanic	1
White	16
Marital status	
Divorced	1
Married	15
Partnered	2
Single	2
Smoking	
Former	3
Never	17
Tumor markers	
ER+	1
HER2/Neu+	1
ER/PR+	7
ER/HER2/Neu+	1
ER/PR/HER2/Neu+	2
Triple-negative	4
Cancer type	
IDC	11
DCIS	2
ILC	2
IMC	1
ALH	1
TNM staging	
Tis	1
T1	12
T2	3
N0	14
N1	1
N2	1
M0	16
BRCA	5
Neoadjuvant chemotherapy	6
Radiation to tissue expanders	1

The most common tumors were ER/PR+ (43.8%) with three being triple-negative (25%). Indications for surgery were invasive cancer (n = 14), ductal carcinoma in situ (n = 2), atypical lobular hyperplasia (n = 1), and prophylactic due to BRCA mutation (n = 3). Of our patient cohort, six patients received neoadjuvant chemotherapy and one received radiation after TE placement. Intraoperative data were collected in Table 2.

**Table 2:** Intraoperative details TE: Tissue expander; ADM: Acellular dermal matrix.

Variable	Median	IQR
Surgery time (min)	213.5	(191.5, 236.8)
Number of reconstructed breasts	39	
Number of total mastectomies	12	
Skin-sparing mastectomies	2	
Nipple-sparing mastectomies	25	
Number of sentinel lymph node biopsies	13	
Left mastectomy weight (g)	468	(305, 635)
Right mastectomy weight (g)	645	(388, 700)
Average TE size (cc)	450	(400, 500)
225 cc	1	
300 cc	1	
375 cc	2	
400 cc	6	
500 cc	5	
600 cc	1	
TE type		
Air	12	
Saline	8	
Average intraoperative TE volume (cc)	100	(93.8, 124.2)
Average ADM area (cm^2^)	240	(240, 240)
Left final volume (cc)	350	(225, 400)
Right final volume (cc)	375	(225, 400)
Time to left TE replacement (days)	152	(99, 189)
Time to right TE replacement (days)	151.5	(83, 180)

**Figure 4:**
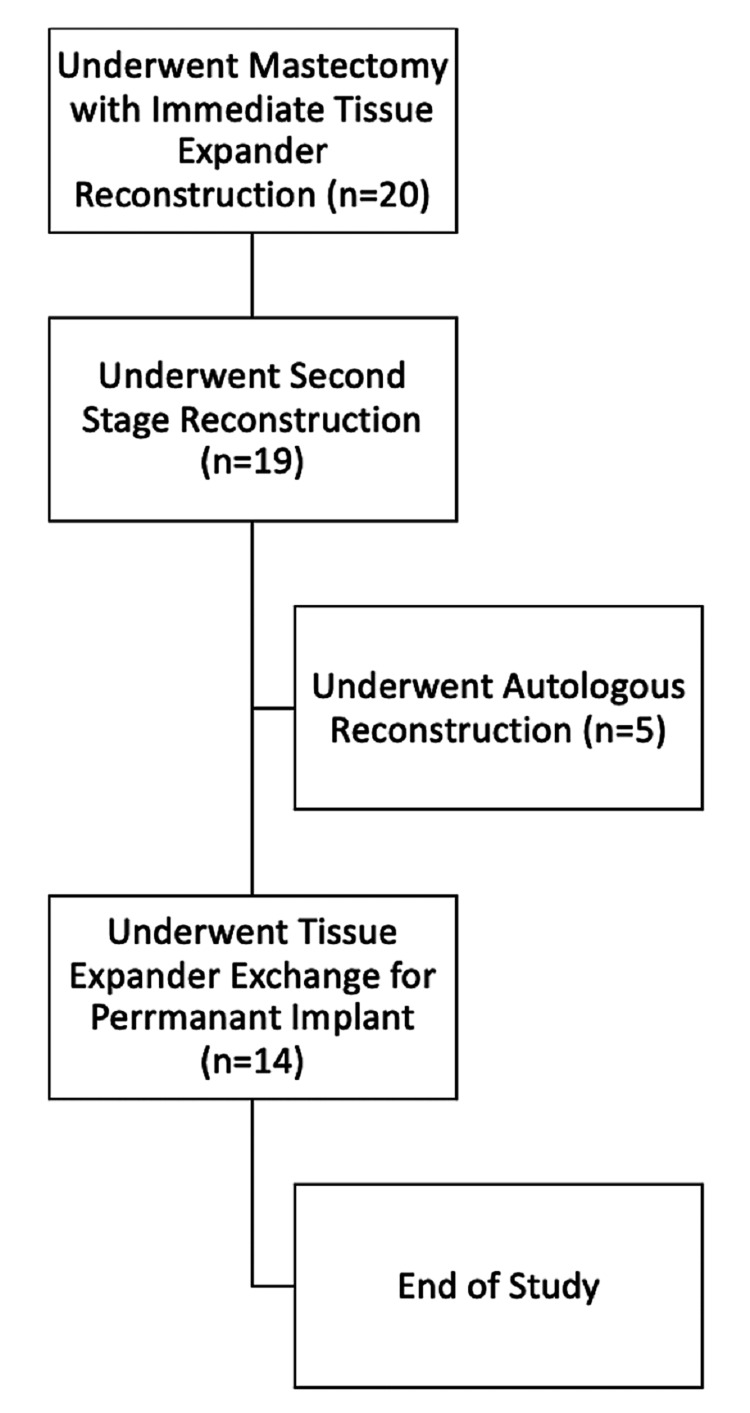
Flowchart of the patient cohort during the time of study

Most patients received a 400 cc TE (37.5%) that was filled with air (60%). Mastectomy followed by immediate prepectoral TE placement with AlloDerm® was performed on bilateral breasts (n = 18) and unilaterally on the right breast (n = 1). For the second stage of reconstruction, 13 patients underwent TE exchange for a permanent implant (Figure 4).

**Figure 5:**
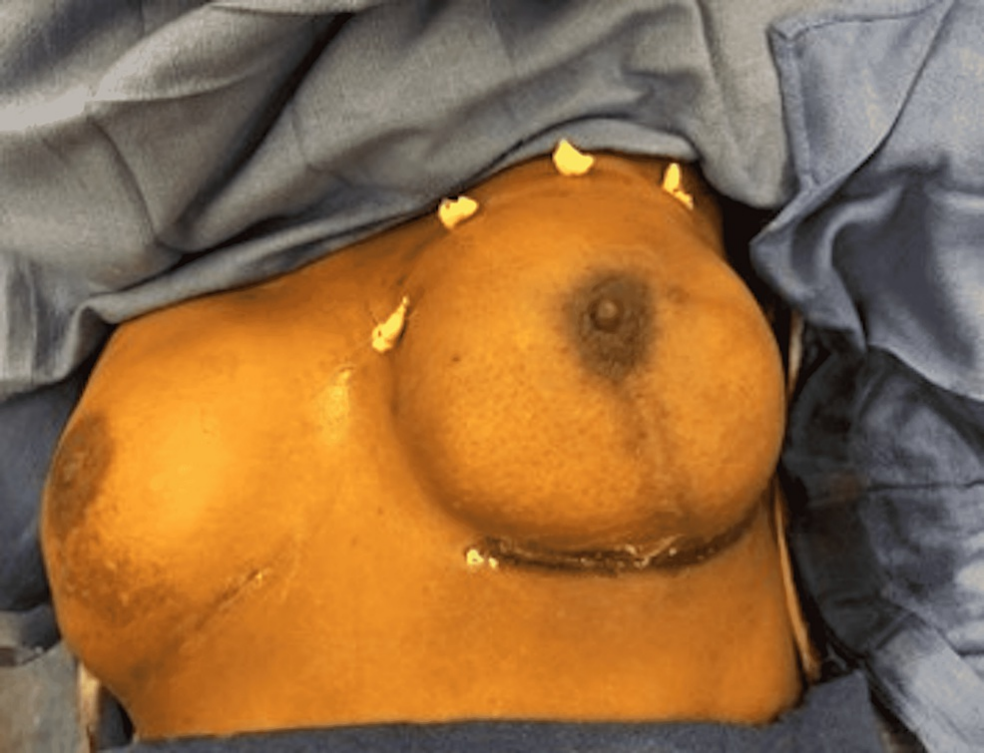
Intraoperative view of breasts after tissue expander-to-implant exchange

One patient did not complete preoperative survey data but completed surveys through the study endpoint. The final outcomes after TE-to-implant exchange are shown in Figure 5.

Pain VAS, Patient Pain Assessment Questionnaire, Subjective Pain Survey, and BPI-SF pain interference

**Figure 6:**
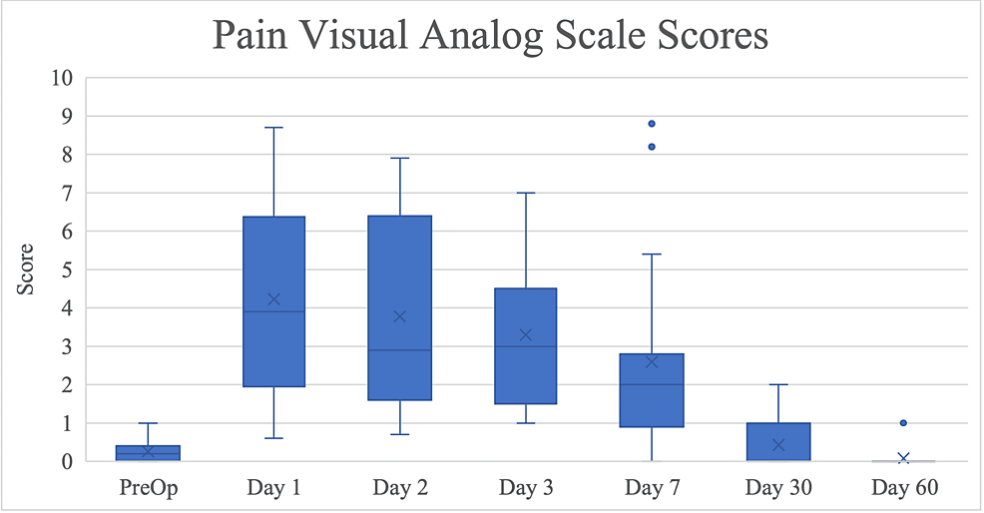
Pain visual analog scale (VAS) scores by time point of follow-up, presented as median with interquartile range. Time points range from initial preoperative consult to 60 days after immediate reconstruction with TE placement in the prepectoral plane with AlloDerm® reinforcement.

Pain VAS scores decreased to 50% of their postoperative day 1 values on day 7 and returned to preoperative baseline by 30 days after TE placement (Figure 6).

The highest pain scores from the Patient Pain Assessment Survey were recorded on postoperative day 1, during which the median worst pain in the last 24 hours was 6.0 (IQR 5.0, 7.8) and the least pain in the last 24 hours was 2.0 (IQR 1.0, 2.0) (Table 3).

**Table 3:** Pain VAS, Patient Pain Assessment Questionnaire, BPI-SF pain interference, and static and moving pain scores VAS: Visual analog scale; BPI-SF: Brief Pain Inventory-Short Form.

	Pre-op			Day 1			Day 2			Day 3			Day 7			Day 30			Day 60		
	n	Med	IQR	n	Med	IQR	n	Med	IQR	n	Med	IQR	n	Med	IQR	n	Med	IQR	n	Med	IQR
Pain VAS	19	0.2	(0.00-0.40)	20	3.9	(1.95-6.34)	19	2.9	(1.60-6.40)	17	3.1	(1.35-4.55)	19	2.0	(0.9-2.8)	14	0.3	(0.0-0.77)	13	0.1	(0.0-0.24)
BPI	18	0.0	(0.00-0.32)	20	4.2	(1.79-5.53)	20	3.9	(1.71-5.96)	20	3.0	(0.87-4.14)	20	2.6	(0.89-4.0)	14	0.3	(0.0-1.93)	13	0.0	(0.0-0.21)
Nausea	19	0.0	(0.00-0.00)	20	0.0	(0.00-1.75)	20	0.0	(0.00-0.00)	20	0.0	(0.00-0.00)	20	0.0	(0.00-0.75)	15	0.0	(0.00-0.00)	13	0.0	(0.00-0.00)
Vomiting	19	0.0	(0.00-0.00)	20	0.0	(0.00-0.00)	20	0.0	(0.00-0.00)	20	0.0	(0.00-0.00)	20	0.0	(0.00-0.00)	15	0.0	(0.00-0.00)	13	0.0	(0.00-0.00)
Average pain in the last 24 hours	19	1.0	(0.00-2.00)	20	3.0	(3.00-6.50)	20	4.0	(2.25-5.00)	19	3.0	(3.00-4.00)	20	2.5	(1.25-4.00)	15	1.0	(0.00-2.00)	13	1.0	(0.00-1.00)
Pain at the time of survey	19	0.0	(0.00-1.00)	20	3.0	(2.00-4.75)	20	3.0	(1.25-4.75)	20	2.0	(1.00-4.00)	20	2.0	(1.00-4.00)	15	1.0	(0.00-1.00)	13	0.0	(0.00-0.50)
Worst pain in the last 24 hours	19	1.0	(0.00-2.00)	20	6.0	(5.00-7.75)	20	5.5	(4.00-7.00)	20	5.0	(3.00-5.75)	20	3.5	(2.00-6.75)	15	2.0	(0.00-3.00)	13	1.0	(0.00-2.00)
Least pain in the last 24 hours	19	0.0	(0.00-0.00)	20	2.0	(1.00-2.00)	20	1.5	(1.00-3.00)	20	1.0	(0.00-3.00)	20	1.0	(0.00-2.00)	15	0.0	(0.00-1.00)	13	0.0	(0.00-0.00)
Lying in bed	19	0.0	(0.00-0.00)	20	1.5	(0.00-8.50)	19	3.0	(0.00-5.00)	20	2.5	(0.00-7.00)	20	1.5	(0.00-5.25)	15	1.0	(0.00-2.00)	13	0.0	(0.00-1.00)
Sitting up in bed	19	0.0	(0.00-0.00)	20	3.5	(1.00-5.00)	20	4.0	(2.00-6.00)	20	2.5	(0.25-4.00)	20	1.0	(0.00-3.75)	15	0.0	(0.00-1.00)	13	0.0	(0.00-0.00)
Laughing	19	0.0	(0.00-0.00)	20	2.5	(1.00-5.75)	20	2.0	(1.00-6.00)	20	1.5	(0.00-4.00)	20	1.0	(0.00-3.75)	15	0.0	(0.00-0.00)	13	0.0	(0.00-0.00)
Coughing	19	0.0	(0.00-0.00)	19	3.0	(2.00-6.00)	20	3.0	(1.00-6.75)	20	2.0	(0.00-5.00)	20	1.5	(0.00-4.75)	15	0.0	(0.00-1.00)	13	0.0	(0.00-0.00)
Sneezing	19	0.0	(0.00-0.00)	15	2.0	(0.00-7.00)	18	2.5	(0.00-7.25)	17	1.0	(0.00-6.00)	18	1.0	(0.00-5.25)	15	0.0	(0.00-1.00)	13	0.0	(0.00-0.00)
Talking	19	0.0	(0.00-0.00)	20	0.5	(0.00-1.75)	20	0.0	(0.00-2.00)	20	0.0	(0.00-1.75)	20	0.0	(0.00-0.00)	15	0.0	(0.00-0.00)	13	0.0	(0.00-0.00)
Watching TV	19	0.0	(0.00-0.00)	20	0.0	(0.00-1.00)	20	0.0	(0.00-1.00)	20	0.0	(0.00-0.75)	19	0.0	(0.00-1.00)	15	0.0	(0.00-0.00)	13	0.0	(0.00-0.00)
Getting out of bed	19	0.0	(0.00-0.00)	20	6.0	(4.00-7.75)	20	6.0	(3.00-7.75)	20	3.0	(1.25-5.75)	20	2.0	(2.00-6.50)	15	0.0	(0.00-1.00)	13	0.0	(0.00-1.00)
Bending	19	0.0	(0.00-0.00)	20	5.0	(2.00-9.00)	20	5.0	(2.25-7.75)	20	3.0	(2.00-5.75)	20	3.0	(1.00-5.50)	15	0.0	(0.00-2.00)	13	0.0	(0.00-0.50)
Bathing yourself	19	0.0	(0.00-0.00)	14	7.5	(4.25-10.00)	17	7.0	(2.50-7.50)	19	3.0	(2.00-7.00)	18	3.0	(1.00-5.50)	15	1.0	(0.00-1.00)	13	0.0	(0.00-0.00)
Dressing yourself	19	0.0	(0.00-0.00)	19	5.0	(4.00-8.00)	20	5.5	(2.25-7.00)	20	3.0	(1.25-6.00)	19	3.0	(1.00-5.00)	15	1.0	(0.00-2.00)	13	0.0	(0.00-0.50)
Lifting	19	0.0	(0.00-0.00)	14	7.5	(3.75-10.00)	18	8.0	(4.50-10.00)	18	5.0	(1.75-9.25)	17	4.0	(2.00-9.50)	15	2.0	(0.00-3.00)	13	1.0	(0.00-1.50)
Carrying groceries	19	0.0	(0.00-0.00)	11	10.0	(6.00-10.00)	15	10.0	(5.00-10.00)	15	8.0	(0.00-10.00)	16	5.5	(1.00-10.00)	15	1.0	(0.00-4.00)	13	0.0	(0.00-0.50)
Climbing stairs	19	0.0	(0.00-0.00)	15	2.0	(1.00-5.00)	20	2.0	(0.00-4.75)	20	1.0	(0.00-2.00)	19	0.0	(0.00-2.00)	15	0.0	(0.00-0.00)	13	0.0	(0.00-0.00)
Stretching	19	0.0	(0.00-1.00)	20	5.5	(2.50-8.00)	20	5.5	(2.25-8.00)	20	2.5	(1.25-5.00)	20	2.0	(1.00-6.50)	15	1.0	(0.00-3.00)	13	0.0	(0.00-1.00)
General activity	19	0.0	(0.00-0.00)	20	6.5	(4.25-7.75)	20	5.0	(1.25-8.00)	20	5.0	(2.25-7.00)	20	2.5	(1.25-6.50)	15	0.0	(0.00-2.00)	13	0.0	(0.00-0.00)
Mood	19	0.0	(0.00-0.00)	20	1.5	(1.00-4.50)	20	2.5	(0.00-4.75)	20	1.5	(0.00-3.00)	20	1.5	(0.00-3.75)	15	0.0	(0.00-2.00)	13	0.0	(0.00-0.00)
Relations with other people	19	0.0	(0.00-0.00)	19	0.0	(0.00-3.00)	20	0.0	(0.00-3.00)	20	0.5	(0.00-3.00)	20	1.0	(0.00-3.00)	15	0.0	(0.00-1.00)	13	0.0	(0.00-0.00)
Sleep	19	0.0	(0.00-0.00)	20	4.0	(1.25-6.75)	20	2.0	(1.00-6.00)	20	1.0	(0.25-5.00)	20	1.5	(0.00-4.75)	15	0.0	(0.00-2.00)	13	0.0	(0.00-1.50)
Enjoyment of life	19	0.0	(0.00-0.00)	20	1.0	(0.00-7.00)	20	1.5	(0.00-6.75)	20	2.0	(0.00-4.75)	20	2.5	(0.00-5.00)	15	0.0	(0.00-2.00)	13	0.0	(0.00-0.00)
Ability to concentrate	19	0.0	(0.00-0.00)	20	4.5	(1.25-6.00)	20	1.5	(1.00-5.75)	20	1.0	(0.00-4.00)	20	1.0	(0.00-4.00)	15	0.0	(0.00-2.00)	13	0.0	(0.00-0.00)
Appetite	19	0.0	(0.00-0.00)	20	1.0	(0.00-6.00)	20	1.5	(0.00-5.00)	20	1.0	(0.00-3.75)	20	0.5	(0.00-3.75)	15	0.0	(0.00-0.00)	13	0.0	(0.00-0.00)
Overall quality of life	19	0.0	(0.00-1.00)	20	2.5	(1.00-6.00)	20	3.5	(0.25-6.00)	20	2.0	(0.00-4.00)	20	2.5	(0.25-5.00)	15	0.0	(0.00-2.00)	13	0.0	(0.00-0.50)

**Figure 7:**
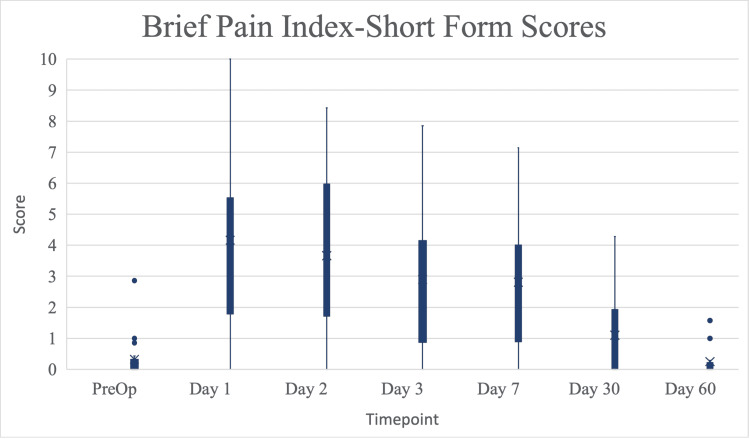
Scores on the BPI-SF by time point of follow-up, presented as median with interquartile range. Time points range from initial preoperative consult to 60 days after immediate reconstruction with TE placement in the prepectoral plane with AlloDerm® reinforcement. BPI-SF: Brief Pain Inventory-Short Form; TE: Tissue expander.

Patient-reported static pain scores were highest on post-op day 1 for “Sitting up in bed” (median = 3.5, IQR 1.0, 5.0). Moving pain scores showed the highest score on postoperative day 1 for “Carrying groceries” (median = 10.0, IQR 6.0, 10.0). All static and moving pain scores progressively declined to preoperative values by day 60. The mean subjective pain score was 3.0 (0.5 standard deviation). None of the seven patients scoring outside the standard had a history of anxiety or depression. BPI-SF pain interference scores followed a similar trend to pain VAS (Figure 7).

PONV scores and opioid use

Median PONV scores remained at 0 from postoperative day 0 to day 7 (Table 4).

**Table 4:** Median PONV scores post-TE insertion day 0 to day 7 PONV: Postoperative nausea and vomiting; TE: Tissue expander.

	Day 0		Day 1		Day 2		Day 3		Day 7	
	Median	IQR	Median	IQR	Median	IQR	Median	IQR	Median	IQR
PONV score	0	(0-1.3)	0	(0-0)	0	(0-0.3)	0	(0-0)	0	(0-0)

Opioid use decreased across all participants over the postoperative period. From our patient cohort, opioid use was reported on post-TE placement day 1 (n = 17) and decreased on day 7 (n = 10). By day 30, opioid use was still reported (n = 2). One patient reported pain bilaterally at the axillae, lower ribs, and left medial arm and forearm; the other reported pain at the sternoclavicular joint and right axilla. By day 60, only one patient reported opioid use without specifying her pain location (Table 5).

**Table 5:** Number of patients reporting pain medication usage after the tissue expander placement

Pain Medication	n	Pre-op	Day 1	Day 2	Day 3	Day 7	Day 30	Day 60
Hydromorphone	19	0	0	0	0	1	0	0
Dilaudid	19	0	0	0	0	1	0	0
Percocet	19	0	1	0	0	1	0	0
Oxycodone	19	0	8	9	6	4	1	1
Tramadol	19	0	8	5	5	3	1	0
Gabapentin	19	0	12	14	13	9	1	0
Valium	19	0	5	2	2	2	1	0
Tylenol	19	0	17	16	16	15	6	4
Celebrex	19	0	5	4	4	4	1	0
Flexeril	19	0	1	3	3	1	1	0
Ibuprofen	19	0	0	1	1	3	2	0

Most participants (n = 19) reported non-opioid analgesic use, most notably gabapentin and acetaminophen (Tylenol), through day 30.

Quality of life: BREAST-Q and SF-36

**Figure 8:**
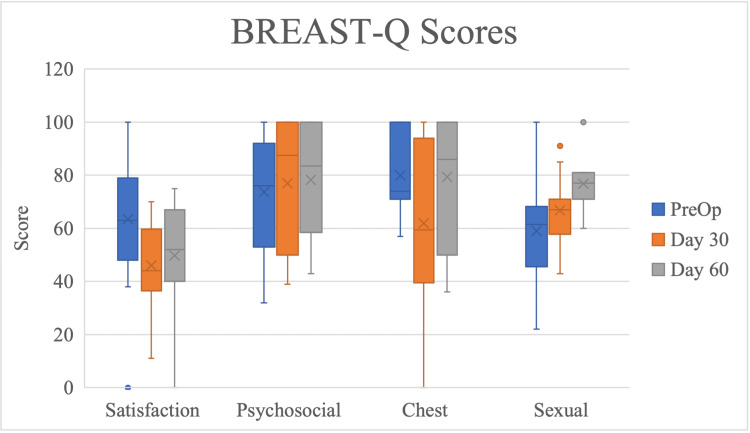
Scores on BREAST-Q subscales by time point of follow-up, presented as median with interquartile range. Time points range from initial preoperative consult to 60 days after immediate reconstruction with TE placement in the prepectoral plane with AlloDerm® reinforcement. TE: Tissue expander.

Changes in BREAST-Q scores for satisfaction with breasts, psychosocial well-being, and physical well-being: Chest was not significantly different across time points. BREAST-Q sexual well-being scores significantly increased from preoperative to postoperative day 60 time points (Figure 8, Table 6).

**Table 6:** BREAST-Q and SF-36 scores SF-36: Short-form 36.

Measure	Scale	p-value
Breast-Q	Satisfaction with breasts	0.061
	Psychosocial	0.86
	Physical well-being: chest	0.069
	Sexual well-being	0.026
SF-36	Physical functioning	<0.001
	Physical limitations	<0.001
	Emotional limitations	0.132
	Energy/fatigue	0.878
	Emotional well-being	0.001
	Social functioning	<0.001
	Pain	<0.001
Non-parametric Kruskal-Wallis p-values		

**Figure 9:**
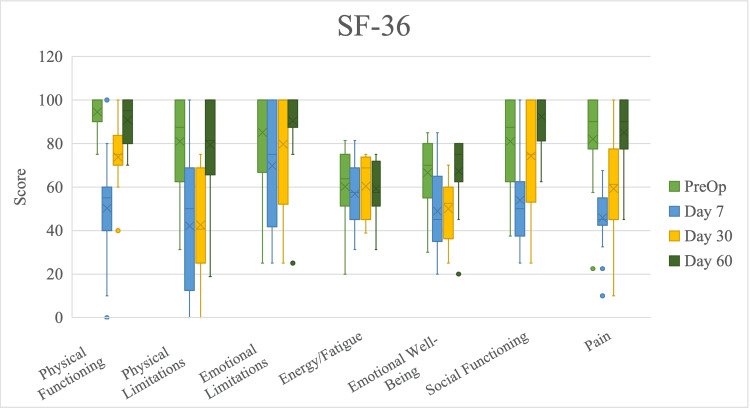
Scores on individual short form-36 (SF-36) domains by time point of follow-up, presented as median with interquartile range. Time points range from initial preoperative consult to 60 days after immediate reconstruction with TE placement in the prepectoral plane with AlloDerm® reinforcement.

SF-36 emotional limitations and energy/fatigue scores did not differ significantly across time points (Figure 9, Table 6).

SF-36 physical functioning scores significantly decreased from preoperative to postoperative day 7 time points and increased from postoperative day 7 to postoperative day 30. SF-36 physical limitations scores decreased nonsignificantly from preoperative to postoperative day 7 time points, did not change from postoperative day 7 to day 30, and increased from postoperative day 30 to day 60; SF-36 emotional well-being scores followed similarly. SF-36 social functioning scores significantly decreased from preoperative to postoperative day 7 time points and increased from postoperative day 7 to day 60; SF-36 pain scores followed similarly.

30- and 60-day complications

One participant (5.6%) experienced an adverse event within 30 days postoperatively. This participant was admitted for an infected left breast seroma. Treatment included IV antibiotics and ultrasound-guided drainage. Two participants (11.1%) experienced a serious adverse event within 30 days postoperatively. The first participant developed left breast cellulitis with wound cultures growing *Serratia marcesens* (Table 7).

**Table 7:** Major and minor postoperative complications NAC: Nipple-areolar complex; SSI: Surgical site infection; TE: Tissue expander.

Tissue Expander		Day 30	Day 60	Implant		Day 30	Day 60
	n	Count	Count		n	Count	Count
Dehiscence	19	0	1	Dehiscence	13	0	0
Hematoma	19	0	0	Hematoma	13	0	0
Seroma	19	1	1	Seroma	13	0	0
Breast cellulitis	19	1	0	Breast cellulitis	13	0	0
Fat necrosis	19	0	0	Fat necrosis	13	1	0
Skin flap necrosis	19	0	0	Skin flap necrosis	13	0	0
NAC necrosis	19	0	0	NAC necrosis	13	0	0
Superficial SSI	19	0	0	Superficial SSI	13	0	0
Deep incisional SSI	19	0	0	Deep incisional SSI	13	0	0
Deep space SSI	19	0	0	Deep space SSI	13	0	0
TE exposure	19	0	0	Implant exposure	13	0	0
TE rupture	19	0	0	Implant rupture	13	0	0
TE removal	19	2	2	Implant removed	13	0	0
TE exchange	19	0	0	Implant exchange	13	0	0
Lymphedema	19	0	0	Lymphedema	13	0	0

She was admitted to the hospital for five days, underwent TE removal, and received IV antibiotics. The second participant underwent TE exchange due to recurrent breast seromas. Two participants (11.1%) experienced serious adverse events within 60 days postoperatively. The first participant experienced bilateral wound dehiscence and required bilateral TE removal. The second participant experienced a right breast seroma that required drainage in the clinic and eventual right TE removal. Five patients experienced complications within 60 days post-TE placement, and the complication rate was calculated to be 25%. Three patients underwent TE removal within 60 days postoperatively, and a removal rate was calculated to be 15%. After TE-to-implant exchange, only one patient experienced a complication within 60 days postoperatively.

## Discussion

In this prospective study assessing postoperative pain in patients who underwent unilateral or bilateral mastectomies followed by immediate TE placement in the prepectoral plane with complete AlloDerm® reinforcement, we found that patient-reported pain scores returned to baseline by postoperative day 30. Pain is a crucial factor to control during the recovery period. It is known that many surgeons utilize ADM for lower pole reinforcement in implant- or TE-based reconstruction since they believe ADM reduces postoperative pain, though the evidence is conflicting [14]. However, when comparing pain in prepectoral versus subpectoral TE reconstruction with ADM, Walia et al. showed that their subpectoral TE cohort reported significantly higher postoperative day 1 pain [15]. Additionally, the prepectoral patient cohort of Walia et al. reported a resolution of pain by postoperative day 7, whereas their subpectoral cohort reported pain until postoperative day 30. Though Walia et al. utilized patient-reported pain scores abstracted from the medical records instead of VAS scores, the difference based on the plane of insertion is apparent.

For a direct comparison of pain VAS scores, McCarthy et al. described a mean pain VAS score of 5.5 ± 2.8 (standard deviation) recorded on day 1 after TE insertion in the subpectoral plane with AlloDerm® [16]. Our mean pain VAS score of 4.2 ± 2.6, recorded on day 1 post-prepectoral TE insertion, was lower but not different clinically; this was based on a minimally important difference of two points [16]. McCarthy et al. also described pain VAS scores returning to baseline levels after the expansion was completed, which may be around postoperative day 30. Despite their subpectoral TE placement, the findings of McCarthy et al. were similar to ours, which were unexpected. However, differences in preoperative management may influence patient-reported scores that are reported in the literature.

Patient-reported pain was successfully recorded by our surveys, but previous studies had not reported on static and moving pain. These measures could potentially give an insight into the patient's experience of pain after discharge. Physicians can utilize these scores to advise patients to be aware of certain activities that would elicit the most pain during the recovery period.

Additionally, we report a wide variety of baseline perceptions of pain from our Subjective Pain Survey. Though anxiety and depression have been associated with increased perception of acute pain, this was not the case in our cohort [17]. However, there is an established link between anxiety, depression, and increased postoperative pain, so it is important to account for these comorbidities in patients undergoing breast reconstruction [18]. A strong support system along with appropriate medical management may help minimize postoperative pain [19].

Our study also reports BPI-SF pain interference scores and PONV scores within 60 days after TE placement, which are not commonly described in the literature. One study by Park et al. recorded BPI-SF pain interference and PONV scores after ultrasound-guided blocks of the erector spinae muscles before mastectomy with TE reconstruction in the subpectoral plane [20]. They only report three- and six-month BPI-SF pain interference scores, so we cannot compare them to our 60-day values, but notably, the use of the erector spinae block reduced opioid usage within 24 hours postoperatively. PONV scores of Park et al. were higher than our study potentially due to differential utilization of preoperative anti-emetics [20,21]. Though BPI-SF and PONV scores could not be compared, Park et al. showed that ultrasound-guided paravertebral blocks of the erector spinae muscles may help reduce opioid usage.

When comparing opioid usage between prepectoral versus subpectoral reconstruction, one study has shown a reduction in immediate postoperative utilization of opioids in their prepectoral versus subpectoral cohorts [22]. Van Boerum et al. also showed that patients report opioid use for a mean of 7.4 ± 6.5 (standard deviation) days after being discharged home and that prepectoral placement was associated with lower post-discharge opioid consumption [23]. Though we did not make a comparison to subpectoral reconstruction in our study, these results suggest that the pain in prepectoral reconstruction can mostly be controlled with non-opioid medications, which the majority of our patients took throughout their time in the study. Prescription of opioids is provider-dependent, but with prepectoral reconstruction, the overall number of pills can be reduced, which has been shown to be feasible in breast reconstruction patients [24].

Reduction of opioid usage is important in the recovery period, but overall success can be best defined through patient satisfaction. Previous studies have shown that BREAST-Q scores do not differ significantly across domains in patients receiving immediate, TE-based prepectoral versus subpectoral breast reconstruction [15]. The significant increase in sexual well-being scores from preoperative baseline to day 60 post-TE placement was possibly due to patient adjustment to the presence of the TE and the gradual increase in volume over time. Nelson et al. matched BREAST-Q physical well-being, the chest scores for patients undergoing bilateral prepectoral and subpectoral TE-based breast reconstruction with ADM, and found no significant differences up to 90 days postoperatively [25]. Their cohort’s scores were not clinically different than ours based on a minimally important difference between four previously established studies in the literature [26]. It may be due to prepectoral TE placement having better cosmetic results, but further studies are needed to examine this hypothesis.

Regarding function, SF-36 data showed significant differences between the scores of physical functioning, physical limitations, and pain from preoperative baseline to day 60 post-TE insertion. Though previous studies have shown that postoperative SF-36 summary physical health scores were lower among patients who received prepectoral versus subpectoral TE placement, our study demonstrates the potential for patients to return to their preoperative baseline score by day 60 [15]. Future studies should investigate relative time courses of SF-36 physical functioning, physical limitations, and pain across time points comparing patients who receive prepectoral versus subpectoral breast reconstruction as previously published differences in scores may resolve early in the postoperative course.

Patient-reported outcome scores are effective in determining patient satisfaction and quality of life, but it is also important to address postoperative complications. There is no consensus on whether prepectoral versus subpectoral placement is associated with more complications, but prepectoral has been associated with higher seroma rates [27,28]. Though our TE removal rates were higher than previously reported values, the complication rates in the prepectoral cohort of Haddock et al. were similar to ours [28]. Though the current evidence suggests that certain patients can undergo reconstruction without ADM, longer term, prospective studies are needed for definitive answers [29]. Immediate TE-based reconstruction has additionally been shown to have more complications than direct-to-implant, which was observed in our cohort [30]. Furthermore, it should be known that following a complication requiring TE removal, a second attempt at TE placement before implant exchange is often successful, and there are no specific comorbidities that would preclude a second attempt [31].

This study has several limitations. Although this study had a relatively consistent and thorough follow-up, some questionnaires were incompletely answered resulting in a loss of data. This study originally intended to utilize 3D imaging techniques to capture changes in the postoperative period in prepectoral reconstruction; however, variations in imaging prohibited abstraction of data and inclusion in our final analysis. As a result, postoperative scars were not able to be assessed. We also did not analyze preoperative analgesia by our anesthesia team, which may have confounded pain scores. Finally, this study is descriptive in nature and reports the postoperative time course in pain and opioid use. These findings do not substantially add to the literature, but the prospective, quality of life data presented are valuable data that may aid in future studies.

## Conclusions

This prospective study described postoperative measures of pain VAS scores, Patient Pain Assessment Questionnaires, Subjective Pain Surveys, BPI-SF pain interference scores with pain medication usage, PONV scores, and BREAST-Q and SF-36 survey scores in patients who underwent mastectomies followed by two-stage reconstruction with tissue expansion and implant placement in the prepectoral plane. Our findings show that pain scores return to baseline by postoperative day 30 with 90% stopping opioid use by that time as well. BREAST-Q sexual well-being scores at postoperative day 60 were higher than preoperative scores, which have not been previously reported in the literature. Future studies are required to understand the utility of ADM in TE-based breast reconstruction in the prepectoral plane.
